# Systematic review: Safety and efficacy of atomoxetine in children and adolescents with autism spectrum disorder

**DOI:** 10.1002/jcv2.70022

**Published:** 2025-06-09

**Authors:** Nihit Gupta, Daniel Boyes, Hunter Hanlon‐Taylor, Mayank Gupta

**Affiliations:** ^1^ Dayton Children's Hospital Dayton Ohio USA; ^2^ Wright State University Boonshoft School of Medicine Fairborn Ohio USA; ^3^ Southwood Psychiatric Hospital Pittsburgh Pennsylvania USA

**Keywords:** Asperger's, atomoxetine, autism spectrum disorder, autistic disorder, neurodevelopmental disorders, pervasive developmental disorder not otherwise specified

## Abstract

**Background:**

This systematic review aimed to assess the current evidence on the efficacy and safety of Atomoxetine in common clinical attention‐deficit hyperactivity disorder (ADHD) symptoms in the context of autism spectrum disorder (ASD) for children and adolescents. Some of these common clinical symptoms of ADHD in the context of ASD include core symptoms of ASD, ADHD, depression, anxiety, mood instability/irritability, and cognitive symptoms.

**Methods:**

Major medical literature were searched for randomized controlled trials (RCTs), open‐label trials, and other relevant studies or clinical trials reporting on pediatric (age <18 years) patients with ASD treated with Atomoxetine for any reason. Databases were searched January of 2024 and include PubMed, Google Scholar, Web of Science, Scopus, PsycINFO, and Embase. Exclusion criteria were unpublished data and multiple reports from the same data set.

**Results:**

A total of 100 abstracts were screened, and 16 clinical trials were selected for inclusion. Out of these 16 clinical trials there were two RCTs (*n* = 128 and 97), four open‐label trials (*n* = 24, 12, 12, and 16), eight extension studies (*n* = 128, 97, 88, 97, 97, 117, 128, and 94), one observational study (*n* = 4), and one crossover study (*n* = 16). Meta‐analysis was not performed due to a lack of homogeneity in the two RCTs. There were limited studies available with a need for more high‐power studies. In the current studies, most suggested that Atomoxetine was well tolerated and safe in pediatric patients with ASD. In fact, Atomoxetine response rates were found to be similar to those of methylphenidate in ASD studies, while inducing fewer adverse events and tolerated better.

**Conclusion:**

Further trials are warranted to make conclusive recommendations on Atomoxetine for improvement of common clinical symptoms of ADHD in the ASD pediatric population. Given limited approved therapies for common clinical symptoms of ASD in children and adolescents, Atomoxetine could be used as a safe off‐label option due to a favorable tolerability profile and minimal adverse effects.

## Graphical abstract

This systematic review aimed to assess the current evidence on the efficacy and safety of Atomoxetine in common clinical symptoms of autism spectrum disorder (ASD) for children and adolescents. With limited studies available, there is a need for more high‐power studies for the use of Atomoxetine in children and adolescents with autism spectrum disorder. However, given limited approved therapies for common clinical symptoms of ASD in children and adolescents, Atomoxetine could be used for a safe off‐label option due to a favorable tolerability profile and minimal adverse effects.

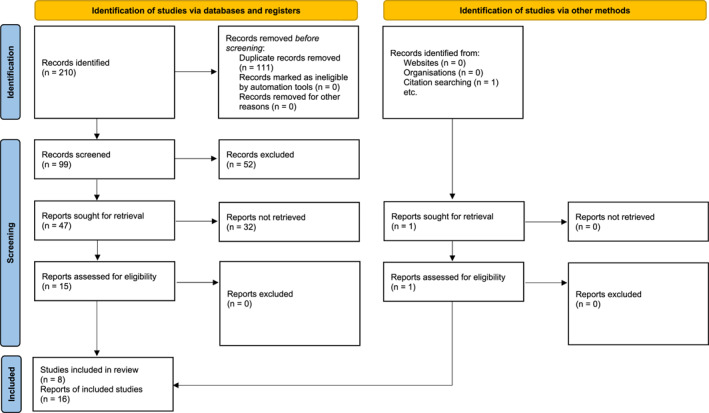

**Key points**

- Atomoxetine may effectively reduce attention‐deficit hyperactivity disorder (ADHD) symptoms in children and adolescents with autism spectrum disorder (ASD), with better tolerability than stimulants like methylphenidate.
- Most studies, though limited, suggest Atomoxetine is safe and shows sustained benefit, but more robust, large‐scale randomized controlled trials are needed.
- Common side effects include decreased appetite, irritability, and mild gastrointestinal symptoms, but serious adverse effects are rare.
- Atomoxetine could be a valuable off‐label option for treating ADHD symptoms in ASD, especially in children who don't respond well to stimulants.

## INTRODUCTION AND BACKGROUND

The Diagnostic and Statistical Manual for Mental Disorder 5th edition (DSM‐V) defines autism spectrum disorder (ASD) as a neurodevelopmental disorder characterized by impairment in communication and socialization and restricted repetitive behaviors and interests (American Psychiatric Association, 2013). Diagnoses such as autism, pervasive developmental disorder (PDD), Rett syndrome, Asperger's syndrome, and childhood disintegrative disorder that previously had been separate diagnoses now fall under the umbrella term of ASD according to the DSM‐V (American Psychiatric Association, 2013). All those separate diagnoses shared core symptoms with different degrees of clinical severity, lending to the rationale of why they were all combined into a diagnosis on a spectrum (Chahboun et al., 2022).

The variability in prevalence of ASD is large with approximately 1/100 children worldwide diagnosed with ASD and 1/36 eight‐year‐old children in the U.S. having ASD (Maenner et al., 2023; Zeidan et al., 2022). ASD is primarily a clinical diagnosis applying DSM‐5 diagnostic criteria based on developmental history and observed behaviors (American Psychiatric Association, 2013). There are interview techniques to assist in making a diagnosis of ASD, such as the Autism Diagnostic Observation Schedule (ADOS) and the Autism Diagnostic Interview‐Revised (ADI‐R) (Lebersfeld et al., 2021). However, ADOS and ADI‐R data has been shown to be less accurate in a clinical setting compared to a research setting (Lebersfeld et al., 2021). With no medical tests for the diagnostics of ASD and many gaps in the screening and diagnostic tools, there are certain challenges that arise when diagnosing ASD.

Although there is no approved treatment for ASD core symptoms, evidence‐based approaches are available to treat co‐occurring conditions and symptoms (e.g., attention‐deficit hyperactivity disorder [ADHD], anxiety, depression), including both pharmacologic and non‐pharmacologic interventions. Treatments that do not require pharmaceuticals include applied behavioral analysis (ABA) therapy and occupational therapy. ABA therapy encourages desired behaviors and discourages undesired ones to enhance certain skills with occupational therapy designed to help improve skills that are used for activities of daily living. In addition, speech and language therapy is a common developmental technique used in conjunction with ABA therapy and occupational therapy which may improve verbal communication or nonverbal communication through mediums such as signs, gestures, or pictures. Many other types of training not mentioned here can also be used based on patient preferences and availability of support. It should be noted, however, that ABA is potentially ineffective due to a lack of long‐term studies (Shkedy et al., 2021; Smith‐Young et al., 2020; Sterman et al., 2023).

For cases where non‐pharmacologic treatment is deemed insufficient, there are hopes for psychopharmacology therapy to help fill the gap for children with ASD (Smith‐Young et al., 2020; Sterman et al., 2023). Currently, there are only two FDA‐approved medications on the market for irritability associated with ASD, Aripiprazole and Risperidone (Blankenship et al., 2010).

A common comorbidity of ASD is ADHD with 50%–70% of individuals with ASD having ADHD (Hours et al., 2022). Until DSM‐5, ASD and ADHD were considered mutually exclusive, and only after 2015 with changes to the DSM‐5 could both disorders be diagnosed together. The presence of ADHD further exacerbates delay in learning when presented with ASD. There is also a difference in prevalence of ASD and ADHD in the genders. ASD and ADHD occur in males more often than females with recent studies showing they occur four and three times more likely in males than females, respectively (Mahendiran et al., 2019). Oftentimes, ASD complicates treatments for various comorbidities, requiring more specialized therapies. While a great deal of literature exists for ADHD treatment in children, little exists for ADHD in children with concomitant ASD. Methylphenidate, amphetamine salts, and other stimulant medications represent first‐line agents for typical developing children due to safety and efficacy (Wolraich et al., 2019). While stimulants provide an improvement in symptoms in 80% of patients with ADHD, a systematic review found poor evidence in a series of crossover trials that methylphenidate may improve inattention and hyperactivity in children affected by ASD (Sturman et al., 2017). In some studies, stimulant medications were found to not be well‐tolerated by children with ASD due to adverse effects frequency (Research Units on Pediatric Psychopharmacology Autism Network, 2005). In a crossover study analyzing the use of methylphenidate for children with ASD and ADHD, 18% of 72 subjects withdrew because of numerous adverse effects (Research Units on Pediatric Psychopharmacology Autism Network, 2005). Other FDA‐approved medications for core symptoms of ADHD in children are non‐stimulants such as atomoxetine, guanfacine, and clonidine (Wolraich et al., 2019).

Atomoxetine, a selective norepinephrine reuptake inhibitor, is of particular interest due to evidence suggesting high efficacy for the treatment of ADHD with comorbid conditions such as anxiety, bipolar disorder, oppositional defiant disorder, ASD, etc. (Clemow et al., 2017). Atomoxetine was first approved by the FDA to treat ADHD in 2002 (Fu et al., 2022). It was the first non‐stimulant medication approved for treatment of ADHD, providing a new alternative in pharmacotherapy for children who do not tolerate or are non‐responsive to stimulants (Fu et al., 2022). Atomoxetine is thought to work by binding strongly to norepinephrine reuptake transporters in the prefrontal cortex and inhibiting reuptake of norepinephrine into the axon terminal (Fu et al., 2022). It also affects reuptake of dopamine in the prefrontal cortex allowing an increase in dopamine and norepinephrine in the synaptic cleft, causing improvement in concentration and various other symptoms of ADHD (Fu et al., 2022). With part of the pathogenesis of ADHD related to a decrease in dopamine and norepinephrine in the prefrontal cortex, Atomoxetine's mechanism of action (MOA) has made it an effective pharmacotherapy option for individuals with ADHD (Fu et al., 2022). Even with the success of Atomoxetine use for ADHD symptoms over the years, there has been a lack of available evidence for use of Atomoxetine for core symptoms of ADHD with comorbid conditions. With ADHD being a common comorbidity of ASD and limited evidence being available on pharmacotherapy for ADHD in children and adolescents with ASD, we wanted to do a review on this subject. Therefore, we reviewed all available evidence on use, effectiveness, and side effects of Atomoxetine in children and adolescents with ADHD and ASD.

## METHOD

Our systematic review was performed according to PRISMA 2020 guidelines. The protocol was registered at PROSPERO: CRD42024491720.

### Data sources searched

**FIGURE 1:**
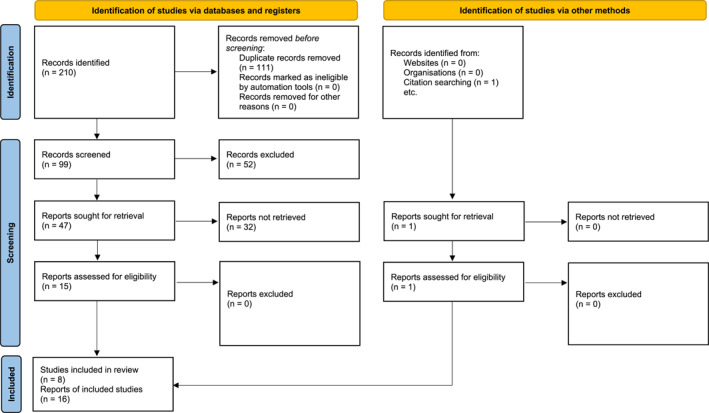
PRISMA flow diagram.

A comprehensive search of several databases from each database's inception to 12/25/2023 was conducted. The databases include PubMed, Google Scholar, Web of Science, Scopus, PsycINFO, and Embase. We also searched for databases of ongoing clinical trials through clinicaltrials.gov. The search was designed by coauthors NG, MG, HT, and DB using controlled vocabulary and keywords: “autism spectrum disorder,” “autistic disorder,” “Asperger's,” “PDD‐NOS,” “neurodevelopmental disorders,” and “Atomoxetine.” We excluded vocabulary such as “pediatrics” from our search per recommendation from our institution's librarian to avoid narrowing of the search for our systematic review. The search was performed in all languages and was limited to human subjects. A manual search of references in included studies was also performed to avoid selection bias. The complete search strategy is available in Figure 1.

### Study selection and eligibility criteria

Three reviewers independently screened titles and abstracts of potentially eligible articles. Subsequently, full texts of eligible articles were reviewed separately by the same reviewers. The study's inclusions criteria were the following:

P: Children under 18 years old with a diagnosis of Autism defined by DSM‐V/IV‐TR/III or ICD‐10 criteria.

I: Studies evaluating use of atomoxetine.

C: Placebo or treatment as usual.

O: Improvement for common clinical symptoms in children and adolescents with ASD including core symptoms (poor social communication and interaction, restricted behaviors and interest, repetitive behaviors, anxiety, and irritability), ADHD symptoms, depression, and cognitive symptoms.

S: Clinical trials.

Exclusion criteria were unpublished data and multiple reports from the same data set (only original research studies were included to prevent unintended duplications of the data set). In case of missing data, we reached out to corresponding authors of the articles for missing information.

### Data collection

Data was extracted from included studies using a standardized data extraction form by the three reviewers (Table 1). Interrater reliability was 99% among the reviewers. We extracted data on the following variables: study characteristics (author, year, study design, sample size, demographic characteristics of participants, inclusions and exclusions criteria), intervention types, outcome measures, and side effects.

**TABLE 1:** Article review summaries.

Article title	Article type	Population	Intervention	Duration (weeks)	Percent of side effects	Effect size	Result
Atomoxetine, parent training, and their combination in children with autism spectrum disorder and attention‐deficit/hyperactivity disorder	RCT	*n* = 128 5–14 years old w/ ASD + ADHD	ATX 1.8 ± PT Placebo ± PT	10	Irritability 42% (ATX ± PT) versus 45% (placebo ± PT) Decreased appetite 47% (ATX ± PT) versus 28% (placebo ± PT) Agitation 28% (ATX ± PT) versus 31% (placebo ± PT) Difficult sleep 30% (ATX ± PT) versus 17% (placebo ± PT) Vomiting 22% (ATX ± PT) versus 16% (placebo ± PT) Constipation 11% (ATX ± PT) versus 14% (placebo ± PT)	ATX alone: 0.68–0.84 PT + placebo: 0.46–0.60 ATX + PT versus placebo: 0.47 ATX versus placebo: 0.64	Atomoxetine and parent training caused a significant improvement in ADHD symptoms compared to the placebo, and atomoxetine (both alone and combined with parent training) was associated with a significant decrease in noncompliance
A randomized double‐blind study of atomoxetine versus placebo for attention‐deficit/hyperactivity disorder symptoms in children with autism spectrum disorder	RCT	*n* = 97 6–17 years old w/ ASD + ADHD	ATX 1.2	8	Nausea 29.2% (ATX) versus 8.2% (placebo) Decreased appetite 27.1% (ATX) versus 6.1% (placebo) Headache 25% (ATX) versus 18.4% (placebo) Fatigue 22.9% (ATX) versus 8.2% (placebo) Upper abdominal pain 18.8% (ATX) versus 6.1% (placebo) Vomiting 14.6% (ATX) versus 10.2% (placebo)	N/A	Results demonstrated a significantly greater improvement in ADHD‐RS scores for the Atomoxetine group than the placebo group
Efficacy of atomoxetine for the treatment of ADHD symptoms in patients with pervasive developmental disorders: A prospective, open‐label study	Open‐label	*n* = 24 8.8 years = mean age w/ PDD + ADHD symptoms	ATX 0.4, 0.8, 1.2	16	Somnolence 8.3% GI malaise 8.3% Irritability 8.3%	N/A	The CGI‐I scale showed significant improvement in 52% of the children and mild improvement in 22% of children, along with statistically significant reduction of variables for attention, conduct problems, and hyperactivity‐impulsivity
Atomoxetine for attention‐deficit/hyperactivity disorder symptoms in children with pervasive developmental disorders: A pilot study	Open‐label	*n* = 12 6–14 years old w/ PDD + ADHD symptoms	ATX 1.19 ± 0.41	10	Anorexia 83% Irritability 75% Sleep problems 58% Drowsiness 33% Nausea 25% Constipation 25%	ADHD RS: 2.3 Connors' Parent RS: 0.19–0.66 Aberrant Behavior Checklist: 0.12–0.44	The atomoxetine group experienced a 44% decrease versus baseline in ADHD symptoms as measured by the ADHD‐RS
Efficacy of atomoxetine in children with severe autistic disorders and symptoms of ADHD: An open‐label study	Open‐label	*n* = 12 5–17 years old w/ severe ASD + ADHD symptoms	ATX 0.25 w/ 0.3–0.4 titration	10	Insomnia 33.33% Decreased appetite 55.56% Moodiness 33.33% Abdominal discomfort 11.11% Irritability 22.22% Diarrhea 11.11%	ABC: Irritability 0.095 Social withdrawal 0.044 Stereotypy 0.097 Hyperactivity 0.086 Inappropriate speech 0.032	According to the ABC screening, there was no statistically significant improvement of irritability, agitation, crying, and hyperactivity rated by the parents
Open‐label atomoxetine for attention‐deficit/hyperactivity disorder symptoms associated with high‐functioning pervasive developmental disorders	Open‐label	*n* = 16 6–14 years old w/ PDD + ADHD symptoms	ATX 0.5, 0.8, 1.2	8	N/A	ABC: 1.0–1.9	12 subjects were rated as “much improved” or “very much improved” on the CGI‐I scale with significant improvement of inattention and hyperactivity‐impulsivity on the ABC and subscales of the SNAP‐IV rated by both parents and teachers
Caregiver satisfaction with a multisite trial of atomoxetine and parent training for attention‐deficit/hyperactivity disorder and behavioral noncompliance in children with autism spectrum disorder	Extension	*n* = 128 5–14.11 years old w/ ASD + ADHD	ATX 1.8 ± PT Placebo ± PT	10	N/A	N/A	87% of respondents reported they would join the study again, 13% said they might, and <1% stated they would not
A randomized, double‐blind comparison of atomoxetine and placebo on response inhibition and interference control in children and adolescents with autism spectrum disorder and comorbid attention‐deficit/hyperactivity disorder symptoms	Extension	*n* = 97 6–17 years old w/ ASD + ADHD	ATX 1.2	8	N/A	N/A	Results demonstrated that atomoxetine improves response inhibition but has no effect on interference control
Long‐term treatment with atomoxetine for attention‐deficit/hyperactivity disorder symptoms in children and adolescents with autism spectrum disorder: An open‐label extension study	Extension	*n* = 88 6–17 years old w/ ASD + ADHD	ATX 1.2	20	Abdominal pain 6.8% (weeks 1–8) versus 2.3% (weeks 12–20) Decreased appetite 9.1% (weeks 1–8) versus 18.2% (weeks 12–20) Early morning awakening 1.1% (weeks 1–8) versus 5.7% (weeks 12–20) Fatigue 6.8% (weeks 1–8) versus 18.2% (weeks 12–20) Headache 14.8% (weeks 1–8) versus 20/5% (weeks 12–20) Nausea 1.1% (weeks 1–8) versus 13.6% (weeks 12–20) Vomiting 5.7% (weeks 1–8) versus 6.8% (weeks 12–20)	N/A	Results demonstrated that inattention, hyperactivity‐impulsivity, and the mean total ADHD‐RS decreased significantly between 8 weeks of initial treatment and the end of the 20 weeks
Atomoxetine in autism spectrum disorder: No effects on social functioning; some beneficial effects on stereotyped behaviors, inappropriate speech, and fear of change	Extension	*n* = 97 6–17 years old w/ ASD + ADHD	ATX 1.2	8	N/A	ABC irritability: 0.2 ABC social withdrawal/lethargy: 0.0 ABC stereotypy: 0.5 ABC hyperactivity: 0.6 ABC inappropriate speech: 0.4 CSBQ total: 0.4	Statistical analysis indicated a significant decrease in ABC subscales of stereotypic behavior, inappropriate speech, and hyperactivity from baseline. Likewise, there was a significant decrease in the CSBQ subscale of fear for changes
No evidence for predictors of response to atomoxetine treatment of attention deficit/hyperactivity disorder symptoms in children and adolescents with autism spectrum disorder	Extension	*n* = 97 6–17 years old w/ ASD + ADHD	ATX 1.2	8	N/A	N/A	There were no significant correlations found between atomoxetine response and baseline characteristics
Atomoxetine and parent training for children with autism and attention‐deficit/hyperactivity disorder: A 24‐week extension study	Extension	*n* = 128 5–14 years old w/ ASD + ADHD	ATX 1.8 ± PT Placebo ± PT	24	N/A	ATX + PT versus ATX for ADHD: 0.54–0.59 ATX + PT versus ATX for Inattention: 0.44–0.68 ATX + PT versus ATX for hyperactivity: 0.41–0.52 ATX + PT versus ATX for ODD: 0.33–0.53	They found that 60% of treatment responders in the initial trial continued to respond well to atomoxetine. 37% of the placebo nonresponders that were started on atomoxetine during the extension study responded well to atomoxetine
Adverse events of atomoxetine in a double‐blind placebo‐controlled study in children with autism	Extension	*n* = 128 5–14 years old w/ ASD + ADHD	ATX 1.8 ± PT Placebo ± PT	10	Constitutional 52% (ATX) versus 53% (placebo) Cardiac 6% (ATX) versus 5% (placebo) GI 77% (ATX) versus 67% (placebo) Neuro 41% (ATX) versus 39% (placebo) Behavioral 80% (ATX) versus 72% (placebo)	N/A	They found that atomoxetine was well tolerated by children with ASD and that the main adverse effects were fatigue, poor appetite, and poor sleep
A 1.5‐year follow‐up of parent training and atomoxetine for attention‐deficit/hyperactivity disorder symptoms and noncompliant/disruptive behavior in autism	Extension	*n* = 128 5–14 years old w/ ASD + ADHD	ATX 1.8 ± PT Placebo ± PT	34	N/A	N/A	According to the SNAP scale and Home Situations Questionnaire, ADHD symptoms and noncompliance decreased slightly from week 34, but overall, was better than baseline at the beginning of the RCT
Observational study with atomoxetine for the treatment of children and adolescents with Asperger's syndrome and ADS	Observational	*n* = 4 6–12 years old w/ Asperger's + ADD	ATX 10 mg/day	52	N/A	N/A	Atomoxetine was shown to be effective in treating the children with high functioning Asperger syndrome and comorbid ADD
Atomoxetine for hyperactivity in autism spectrum disorders: Placebo‐controlled crossover pilot trial	Crossover	*n* = 16 5–15 years old w/ ASD + ADHD symptoms	ATX 2.5	12	N/A	ABC hyperactivity: 0.9 ABC irritability: 0.61 ABC lethargy/social withdrawal: 1.18 ABC stereotypy: 0.87 ABC inappropriate speech: 0.52	An intent to treat analysis showed that on the hyperactivity subscale of the Aberrant Behavior Checklist and nine DSM‐IV ADHD hyperactive/impulsive symptoms, atomoxetine was superior to the placebo

Abbreviations: ABC, Aberrant Behavior Checklist; ADHD, attention‐deficit/hyperactivity disorder; ADHD‐RS, ADHD rating scale; ASD, autism spectrum disorder; ATX, atomoxetine; CGI, clinical global impression; CSBQ, Children's Social Behavior Questionnaire; DSM‐IV, Diagnostic and Statistical Manual for Mental Disorder 4th edition; PDD, pervasive developmental disorder; PT, parent therapy; RCT, randomized controlled trials; SNAP, Swanson, Nolan, and Pelham scale.

### Methodological quality and risk of bias assessment

**FIGURE 2:**
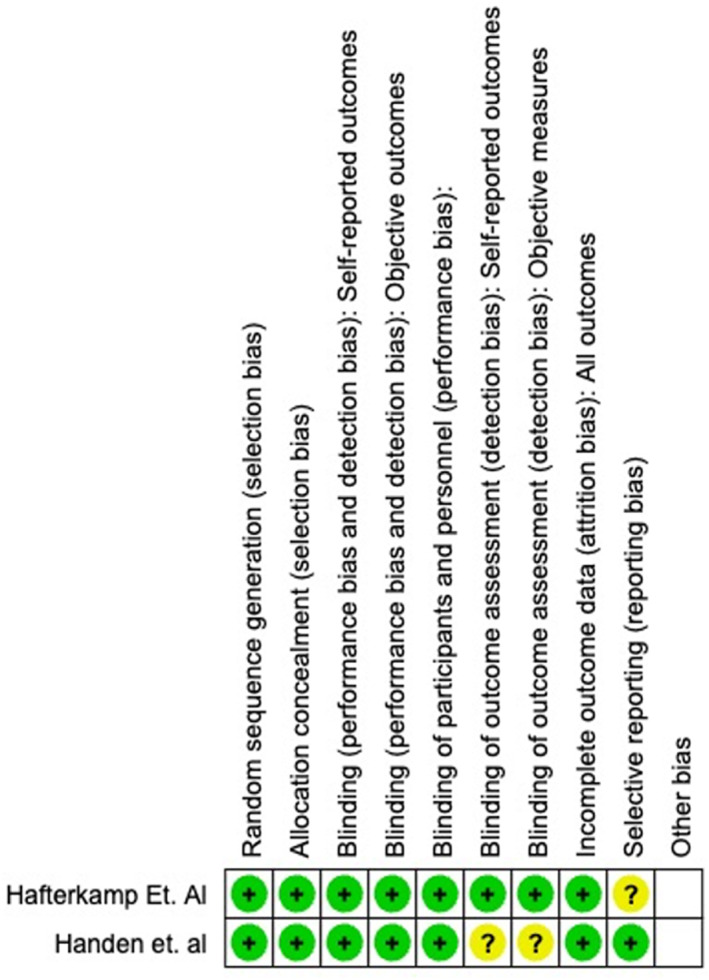
Risk of bias summary. Presents all the judgments made in evaluating the quality in a cross‐tabulation of studies in the review.

**FIGURE 3:**
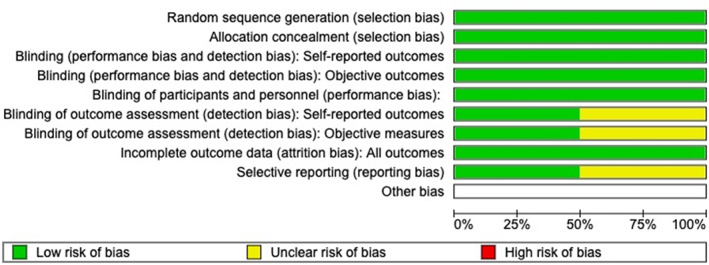
Risk of bias graph. Illustrates the proportion of studies with each of the judgments (“low risk,” “high risk,” and “unclear risk” of bias) for each entry in the systematic review.

We used the Revised Cochrane risk‐of‐bias tool for randomized trials for assessing the risk of bias to evaluate the methodological quality of randomized controlled trial (RCTs) (Sterne et al., 2019). We performed a risk‐of‐bias assessment on all high‐quality studies that were being considered to be included in a meta‐analysis. We deemed all RCTs that met our inclusion criteria to be high‐quality studies that could be considered for a meta‐analysis. We assessed risk of bias for random sequence generation, allocation concealment, blinding of participants and personnel, blinding of outcome assessment, incomplete outcome data, selective reporting, and other biases (Figures 2 and 3). We also included open‐label studies, extension studies, an observational study, and a crossover study that met our inclusion criteria to be thorough in our review, however no risk‐of‐bias assessment was performed on these studies due to these studies being more susceptible to bias and lower quality studies that would not be considered for a meta‐analysis.

## REVIEW

### Overview

Sixteen studies were cited in the review spanning a broad range of study characteristics. These include two RCT's, four open‐label studies, eight extension studies, one observational study, and one crossover study. All trials included a mix of males and females. The initial RCT's divided 97–128 patients between atomoxetine and control groups and assessed for differences in Swanson, Nolan, and Pelham (SNAP), Home Situations Questionnaire (HSQ), ADHD‐RS, and Conners' Teacher Rating Scale‐Revised (CTRS‐RS; Handen et al., 2015; Harfterkamp et al., 2012). Ages ranged from 5 to 14 years old. Both studies found significant improvements in multiple of the aforementioned primary outcomes. The most common side‐effects included nausea, vomiting, decreased appetite, fatigue, headache, and irritability. Extension studies evaluated increased lengths of treatment, caregiver satisfaction, Children's Social Behavior Questionnaire (CSBQ) and Aberrant Behavior Checklist (ABC) outcomes, parent training outcomes, and predictors of response to atomoxetine therapy (Arnold et al., 2018; Harfterkamp et al., 2013, 2014, 2015; Hollway et al., 2016; Smith et al., 2016; Tumuluru et al., 2017; van der Meer et al., 2013). Efficacy varied among subcategories of variables. The age, sex, and genders of the extension study populations were consistent with those found in the initial RCT's. The open‐label trials had anywhere from 12 to 24 participants, ranging in age from 5 to 17 (Charnsil, 2011; Fernández‐Jaén et al., 2013; Posey et al., 2006; Troost et al., 2006). The main variable of interest in these patients was CGI‐I and ADHD‐RS scores. Scores improved significantly among certain categories and were largely unchanged in others. The main side‐effects noted in these trials were somnolence, GI malaise, and irritability.

### Randomized controlled trials

The first RCT (Handen et al., 2015) was a 10‐week, double‐blind, 2 × 2 trial including 128 patients with ASD and ADHD symptoms between 5 and 14 years old. They were randomized into one of four groups including Atomoxetine only, Atomoxetine and parent training, parent training and placebo, and placebo only. Atomoxetine was adjusted to optimal dosing (capped at 1.8 mg/kg/day) over 6 weeks and maintained for four additional weeks. Nine parent training sessions were provided. Primary outcome measurements were measured by parent‐rated *DSM* ADHD symptoms on the SNAP scale and the HSQ. Results indicated Atomoxetine and parent training caused a significant improvement in ADHD symptoms compared to the placebo, and Atomoxetine (both alone and combined with parent training) was associated with a significant decrease in noncompliance. It was also found that combining parent training and Atomoxetine was not significantly better than Atomoxetine alone. Atomoxetine was found to have a similar response rate to methylphenidate while having fewer adverse effects and a better side effect profile in the ASD population.

The second RCT (Harfterkamp et al., 2012) was an 8‐week, double‐blind trial involving 97 patients with ADHD and ASD between 6 and 17 years old. Subjects were assigned randomly in a 1:1 ratio to receive either placebo or Atomoxetine. Atomoxetine was titrated over 3 weeks to a dose of 1.2 mg/kg/day and maintained for the remainder of the 8 weeks. ADHD‐Rating Scale (ADHD‐RS) scores were obtained at both five‐ and 8‐weeks following initiation of treatment. Results demonstrated a significant improvement in ADHD‐RS scores for the Atomoxetine group than the placebo group. Likewise, the Atomoxetine group indicated a significantly greater CTRS‐RS improvement from baseline relative to the improvement for the placebo group, specifically the hyperactivity domain. No serious adverse effects were reported during the trial. Minor adverse effects affecting the Atomoxetine group included nausea, early morning awakening, and decreased appetite.

### Open‐label studies

The first open label study was (Fernández‐Jaén et al., 2013) a 16‐week, prospective study including 24 patients with PDD, based on DSM‐IV‐TR, and symptoms of ADHD. The mean age of patients was 8.8 years old. All patients were treated with an approximate initial dose of 0.4 mg/kg/day of Atomoxetine the first week, 0.8 mg/kg/day the second week, and 1.2 mg/kg/day the third week. The final dose of atomoxetine was given during the third week and continued throughout the study. Maximum dosing of the study was 60 mg/day. One patient with failed adherence to the methodology recommended was excluded from the study. Atomoxetine was shown to be a useful drug. Out of the 23 patients who completed the 16‐week treatment period, the CGI‐I Scale showed significant improvement in 52% and mild improvement in 22% of children. Statistical analysis also showed a statistically significant reduction of variables for attention, conduct problems, and hyperactivity‐impulsivity per the ADHD‐RS‐IV and short form of the Conners' Scale. Only 5 out of 24 patients had adverse effects. The most common adverse effects were somnolence, gastrointestinal malaise, and irritability. These adverse reactions were temporary in 2 patients, while the 3 remaining patients had to discontinue treatment due to adverse effects.

Another open‐label study (Troost et al., 2006) investigated the impact of Atomoxetine on ADHD symptoms and autism characteristics in children with PDD. The study population included 12 children between 6 and 14 years old with PDD complicated by ADHD symptoms. Study participants were administered 1.19 ± 0.41 mg/kg/day of Atomoxetine for 10 weeks. The primary outcome was changes in the ADHD‐RS, utilizing clinician and parent rating scales. The Atomoxetine group experienced a 44% decrease versus baseline in ADHD symptoms as measured by the ADHD‐RS (*p* < 0.003). The Atomoxetine group also experienced a decrease in the Conners' Parent Rating Scale‐R:S; these included 25% in the subscale “Cognitive Problems” (*p* < 0.028), 32% in “Hyperactivity” (*p* < 0.030); and 23% in “ADHD index” (*p* < 0.023). No changes were observed in other subscales. The study was limited by the low number of participants and the fact that it was neither placebo‐controlled nor blinded. The study population included only subjects who were high functioning, rendering the results less adaptable for the impaired autistic population. No serious adverse events were reported.

The third open label study (Charnsil, 2011) was a 10‐week pilot designed study looking at the efficacy of Atomoxetine in treating symptoms of ADHD in children with severe ASD. The study included 12 children between the ages of 5 and 17 years old with severe ASD, according to the DSM‐IV‐TR, who had ADHD symptoms. Children were started on 0.25 mg/kg/day of Atomoxetine and increased every 4–5 days by 0.3–0.4 mg/kg/day unless side effects were intolerable. Individuals who received cytochrome P450 2D6 inhibitors as part of previous treatment were adjusted by 0.2–0.3 mg/kg/day. Doses did not exceed 1.2 mg/kg/day. Three children withdrew from the study during the titration period due to adverse effects such as abdominal discomfort and irritable mood. Only nine children who completed the study were included in the final analysis. Efficacy of Atomoxetine was determined by the ABC to rate ADHD symptoms at baseline, 6 weeks, and 10 weeks. According to the ABC screening, there was no statistically significant improvement of irritability, agitation, crying, and hyperactivity rated by the parents. The CGI‐I scores rated by a nurse showed improvement in the children.

The last open label study (Posey et al., 2006) was an 8‐week prospective study with 16 subjects between 6 and 14 years old looking at efficacy of Atomoxetine for ADHD symptoms in children with PDD. Atomoxetine was started at 0.5 mg/kg/day for a week, increased to 0.8 mg/kg/day during the second week, and titrated up to 1.2 mg/kg/day in the third week. In subjects who were showing minimal improvement, the dose of atomoxetine was increased to 1.4 mg/kg/day at the end of the fourth week. Twelve subjects were rated as “much improved” or “very much improved” on the CGI‐I scale with significant improvement of inattention and hyperactivity‐impulsivity on the ABC and subscales of the SNAP‐IV rated by both parents and teachers. No significant changes were seen on the Conners' Continuous Performance Test. Atomoxetine was tolerated well by most participants with only two subjects withdrawing due to irritability. Another two participants withdrew due to pill swallowing difficulty.

### Extension studies

One extension study (Hollway et al., 2016) examined the caregiver satisfaction associated with the CHARTS (Children with Hyperactivity and Autism Research Treatment Study) trial. The study was an extension of the trial by Harfterkamp which determined whether ATX ± PT was superior to placebo ± PT. The most common side effects in the initial RCT were irritability, decreased appetite, agitation, difficult sleep, vomiting, and constipation. The extension study administered an 18‐question questionnaire to patients' caregivers about satisfaction with research experience. The results were analyzed for correlations between demographic variables, treatment assignments, and response to treatment. 87% of respondents reported they would join the study again, 13% said they might, and <1% stated they would not. Treatment assignment was not significantly associated with caregiver satisfaction on 17 out of 18 items from the questionnaire but did correlate with caregivers thinking the side effects were important. Some limitations were implicated including nine questionnaires that weren't completed, primarily from patients dropping from the study. Participants with data missing had worse ADHD symptoms and higher defiant/oppositional ratings by caregivers. Personnel and caregivers were aware of the questionnaire, thus possibly modifying their behavior.

Another extension study by van der Meer et al. (2013) elaborated on the initial RCT of 97 patients between 6 and 17 years old. This trial compared effects of Atomoxetine versus placebo on interference control and response inhibition in children with ASD and comorbid ADHD. A ‘go‐no‐go’ task was administered to study participants, during which they had to either press or not press a key within a specific response window following a visual stimulus. Variables of interest included the number of false alarms and missed go signals. Results demonstrated Atomoxetine improves response inhibition but has no effect on interference control. On a psychophysiological level, these findings suggest improved frontal‐striatal functioning via Atomoxetine may suffice to enhance response inhibition, but not interference control due to the required parietal‐temporal contributions.

Another extension study (Harfterkamp et al., 2013) following the original study by Harfterkamp et al. looked at long‐term effects of Atomoxetine on children with ASD and comorbid ADHD. This study included 88 patients between 6 and 17 years old who were administered 1.2 mg/kg/day Atomoxetine for 20 weeks as a follow‐up to an 8‐week double‐blind placebo‐controlled period. The endpoint was the ADHD Rating Scale. Results demonstrated that inattention, hyperactivity‐impulsivity, and mean total ADHD‐RS decreased significantly between 8 weeks of initial treatment and the end of 20 weeks. Limitations included absence of a control group, making it difficult to distinguish between natural course versus true treatment effect. The trial did include teacher‐based ratings but focused merely on ADHD symptoms rated by clinicians. Finally, the trial participant group had only a few subjects in the low IQ range, rendering results less generalizable to the entire ASD population.

An extension study of the trial by Harfterkamp et al. (2014) investigated impacts on multiple secondary outcomes including the CSBQ and ABC in the initial population of 97 children. Questionnaires were administered at baseline and following the 8‐week trial. Statistical analysis indicated a significant decrease in ABC subscales of stereotypic behavior, inappropriate speech, and hyperactivity from baseline. Likewise, there was a significant decrease in the CSBQ subscale of fear for changes. None of the other subscales were significantly changed from baseline. A limitation of the study was the choice of rating scales to judge improvements in symptoms. Results relied solely on parent ratings, rather than any clinical assessment or teacher ratings.

Another extension study by Harfterkamp et al. (2015) analyzed predictors of response to Atomoxetine for the patient group in both the original study and 20‐week extension study. ANOVA was utilized to look for correlations between Atomoxetine response and baseline characteristics including IQ, age, sex, CYP2D6 genotype, and type of ASD. There were no significant correlations found between Atomoxetine response and baseline characteristics. However, it was found despite patients with an early response remaining stable in weeks five through eight (*p* = 0.21), patients who did not develop an early response experienced a significant decrease in total ADHD‐RS score between weeks five and eight (*p* = 0.003). No serious adverse events were reported. Although no correlations were observed between Atomoxetine response and patient demographics, evidence from the study suggests clinicians should allow sufficient time before dismissing Atomoxetine as ineffective in the ASD population.

Another extension study (Smith et al., 2016) was of Handen's 10‐week, double blind, 2 × 2 trial looking at using Atomoxetine and parent training for children with ASD and ADHD symptoms. This was a 24‐week extension that looked at how children with ASD and ADHD symptoms who had responded well to Atomoxetine and parent training would continue to respond to treatment through 34 weeks and whether the placebo non‐responders would improve after receiving Atomoxetine. They also wanted to look at if parent training groups had significantly improved on behavioral noncompliance compared to non‐parent training groups over the course of the extension study. They found that 60% of treatment responders in the initial trial continued to respond well to atomoxetine. 37% of the placebo non‐responders that were started on atomoxetine during the extension study responded well to Atomoxetine. Atomoxetine was tolerated well, and adverse effects were comparable to the initial 10‐week study. Lastly, they found that children receiving Atomoxetine and parent training together had better response for their ADHD symptoms than Atomoxetine alone.

Another extension study (Tumuluru et al., 2017) of Handen's 10‐week, double blind, 2 × 2 trial looking at using Atomoxetine and parent training for children with ASD and ADHD symptoms. This study looked specifically at adverse effects in the original Handen double blind, RCT. They found that Atomoxetine was well tolerated by children with ASD and the main adverse effects were fatigue, poor appetite, and poor sleep.

The last extension study (Arnold et al., 2018) we looked at was another extension study of Handen's 10‐week, double blind, 2 × 2 trial looking at using Atomoxetine and parent training for children with ASD and ADHD symptoms. This was a 1.5 years follow up with the participants of the 24‐week extension study. Ninety‐four subjects out of the original 128 participated in this follow up. A fourth of participants had stopped all medication and only a third had continued the study‐initiated Atomoxetine. According to the SNAP scale and HSQ, ADHD symptoms and noncompliance decreased slightly from week 34, but overall, were better than baseline at the beginning of the RCT.

### Observational study

We found one observational study (Gehrmann, 2008) done in Germany that looked at using Atomoxetine in adolescents that had Asperger's and ADD (attention‐deficit disorder). This study involved four participants between 6 and 12 years old that were diagnosed with Asperger's according to the ADI‐R and ADD according to the DSM‐IV. Atomoxetine was started at 10 mg/day in each participant with weekly dosage increases. Comedication with methylphenidate was added to two patient's regimens due to individual needs. Treatment duration varied for each participant with an average duration of 52 weeks for the group. Atomoxetine was shown to be effective in treating children with high functioning Asperger's Syndrome and comorbid ADD. They found the onset of effects from Atomoxetine took about 6–8 weeks to occur and did not find any significant adverse effects.

### Crossover study

The last study we looked at was a double blind, placebo controlled, crossover pilot study (Arnold et al., 2006) that looked at efficacy of Atomoxetine for children with ASD and ADHD symptoms. Sixteen children between 5 and 15 years old with ASD and ADHD symptoms were randomly assigned to order in a crossover of Atomoxetine and placebo, 6 weeks for each trial with a week washout period between trials. Atomoxetine was started at 0.25 mg/kg/day and increased every 4–5 days by increments of 0.3–0.4 mg/kg/day if tolerated. The maximum daily dosage was 1.4 mg/kg/day. Individual's taking a CYP2D6 inhibitor dose were increased by 0.2–0.3 mg/kg/day and capped at 1.2 mg/kg/day. An intent to treat analysis showed on the hyperactivity subscale of the ABC and nine DSM‐IV ADHD hyperactive/impulsive symptoms, Atomoxetine was superior to placebo. However, on nine DSM‐IV ADHD inattentive symptoms, Atomoxetine was not significantly better than placebo. Nine of the participants had good outcomes with Atomoxetine and four with the placebo. There was one serious adverse effect in the study. One of the participants was hospitalized due to violence and associated hallucinations. Other than that, adverse effects were mild with appetite suppression and irritability/mood swings being the two most common side effects.

## DISCUSSION

This systematic review focuses on safety and efficacy of Atomoxetine in children with ASD for the treatment of ADHD and potentially associated symptoms and conditions. It critically reviews empirical data that supports its efficacy in treating core symptoms of ADHD in children with ASD and co‐occurring mental health conditions.

This review included two RCTs, four open‐label trials, eight extension studies, one observational study, and one crossover study varying in length ranging from 8 to 20 weeks with studies focusing on treating ADHD symptoms in children with ASD. Overall, the studies included in the systematic review support symptom improvement in children with ADHD in the setting of ASD using Atomoxetine. The studies showed improvement in noncompliance, hyperactivity‐impulsivity, attention, response inhibition, and inappropriate speech to name a few (Fernández‐Jaén et al., 2013; Handen et al., 2015; Harfterkamp et al., 2012). These symptomatic improvements were measured using various standardized scales such as the SNAP, HSQ, ADHD‐RS scale, Conners' Scale, CSBQ, and ABC with medical professionals, parents, and teachers contributing to rating determination depending on the study.

An evaluation of the efficacy and safety of oral Atomoxetine in children and adolescents with ASD lacks high‐powered double‐blind RCTs. Both RCTs concluded that Atomoxetine was beneficial and safe for ADHD symptoms in children and adolescents with ASD, however, this data is still insufficient. In the Handen RCT (Handen et al., 2015), for noncompliance, ATX and ATX plus parent training were both superior to placebo with an effect size of 0.47–0.64 with *p*‐values of 0.03 and 0.0028 respectively (Handen et al., 2015). Meta‐analysis could not be performed due to lack of homogeneity.

The two RCTs used different measurement scales for their primary outcome measurements, therefore results need further scrutiny. The Handen RCT (Handen et al., 2015) used the SNAP scale and HSQ whereas the Harfterkamp RCT (Harfterkamp et al., 2012) used ADHD‐RS scores and CTRS‐R:S to measure effectiveness of Atomoxetine. Both trials had similar target doses of 1.2 mg/kg/day with different interval adjustment rates for each RCT. With only two RCTs existing for Atomoxetine use in children with ASD that are not combinable show the need for additional RCTs comparing Atomoxetine to placebo with similar scales used for primary outcome measurements.

Another important point to address in these studies is patient selection. Most patients in the study included mixed phenotypes, either ASD with ADHD or PDD with ADHD. This bears great clinical significance as the findings translate best to treating children between 6 and 17 years old with a combination of features. Most studies only included higher‐functioning subjects, rendering results less applicable to the low‐functioning population. Along with that, studies have shown evidence suggesting methylphenidate is less effective in treating ADHD symptoms in children with ASD who have a lower IQ compared to a higher IQ (Ventura et al., 2020). There are growing concerns that children and adolescents with ASD who have lower cognitive functioning are often observed to have curved dose response with methylphenidate (Gupta & Gupta, 2023). In many of these phenotypes, worsening of behavior or activation is observed at higher doses (Gupta & Gupta, 2023).

Atomoxetine may be able to fill this need for treatment of children with ASD and a lower IQ once Atomoxetine has sufficient research for children with ASD and various IQ levels.

Many of the studies supported Atomoxetine as a safe option with minimal adverse effects. Common adverse effects reported were appetite suppression, abdominal discomfort, and irritability. One study even stated Atomoxetine had a better side effect profile in children with ASD than psychostimulants (Handen et al., 2015). Another study stated that 81.3% of patients taking Atomoxetine reported adverse effects and 65.3% patients taking a placebo reported adverse effects, however no serious adverse effects were reported (Harfterkamp et al., 2012). There have been some reports of idiosyncratic hepatotoxicity and reversible liver damage from Atomoxetine, however, these reports are rare (Fu et al., 2022; Tumuluru et al., 2017). In the studies we reviewed, there were no reports of hepatotoxicity or liver damage. However, Atomoxetine can cause elevated liver function tests and behavioral activation, mandating careful monitoring in younger children. Studies recommend dosing between 0.8 and 1.2 mg/kg/day, with the possibility of titrating up to 1.8 mg/kg/day in certain cases (Handen et al., 2015). Furthermore, administering a comorbidity assessment is warranted to determine proper treatment from the perspective of side effects.

As stated before, Atomoxetine's proposed MOA is via binding strongly to norepinephrine reuptake transporters in the prefrontal cortex and inhibiting reuptake of norepinephrine into the axon terminal (Fu et al., 2022). It inhibits reuptake of dopamine in the prefrontal cortex allowing an increase in dopamine and norepinephrine in the synaptic cleft, causing improvement in concentration and various other symptoms of ADHD (Fu et al., 2022). However, because of this MOA, Atomoxetine has an increased risk of elevated blood pressure and heart rate, especially when combined with medications that have an increased risk for high blood pressure and tachycardia (Fu et al., 2022). Atomoxetine has a particular MOA that could be of interest in treating individuals with ASD and ADHD due to its ability to help treat hyperactivity, noncompliance, impulsivity, and reduce anxiety.

In the Handen RCT (Handen et al., 2015), our self‐analysis showed the number needed to treat (NNT) for ADHD responders and improvement of noncompliance was 3.64 and 3.61 respectively. The most common adverse effect in the Handen study (Handen et al., 2015) was irritability among all groups. In fact, there was a greater prevalence of irritability in individuals taking a placebo and undergoing PT than individuals taking atomoxetine. Decreased appetite was the adverse effect with the greatest prevalence in the Atomoxetine groups compared to the non‐atomoxetine groups in the Handen study (Handen et al., 2015). Our self‐analysis showed the number needed to harm (NNH) in the Handen study (Handen et al., 2015) for decreased appetite in Atomoxetine use was 5.26. Having a smaller NNT compared to the NNH in this RCT is advantageous for Atomoxetine.

The extension studies of the two RCTs showed that Atomoxetine continued to be beneficial in many participants after the end of the primary studies, lending to the notion that RCTs with a longer trial duration comparing Atomoxetine to a placebo for treatment of common symptoms of ADHD in children with ASD would be desirable. In addition, most of the open‐label trials showed improvement of ADHD symptoms in children with ASD with all of them stating a larger RCT would be beneficial to perform based on their results.

Currently, risperidone and aripiprazole are the only FDA‐approved medications for ASD (Weissman et al., 2016). They are useful in treating irritability, aggression, and self‐injury in individuals with ASD (Weissman et al., 2016). SSRIs and methylphenidate can be used in ASD for anxiety and ADHD symptoms respectively but are not FDA‐approved (Weissman et al., 2016). As for the current management of ASD, medication is most effective when combined with behavioral therapy (Weissman et al., 2016).

Overall, results from current studies indicate Atomoxetine is strictly superior to placebo while producing fewer adverse effects than Methylphenidate. Patients with ASD who fail current FDA‐approved pharmacologic management may be good candidates for Atomoxetine based on results of current studies on Atomoxetine in children and adolescents with ADHD in the context of ASD.

Within the ASD population, it is important to screen for clinical phenotypes requiring optimization of ADHD symptoms. For those who do not respond to or are unsuitable candidates for stimulant medications, Atomoxetine should be considered. Although the supporting evidence is limited, there is a focus on optimal dosing, with variable dosing schedules often leading to a reduction in symptoms.

### Limitations

Although the two RCTs analyzed present a convincing case for use of Atomoxetine in children with ASD who exhibit features of ADHD, there is nonetheless a shortage of data. A future direction to offset this limitation would be orchestration of additional, longer‐in‐duration, double‐blinded RCT's to amass a larger sample size and longer study. Furthermore, many of these studies exclude lower‐functioning participants with ASD and do not dive deep into the many comorbidities that may occur in patients with ADHD and ASD. Neurodiverse patients with multiple comorbidities may have varying side effect profiles and therefore, impact treatment response. Further phenotype specific research would be beneficial in assessing efficacy of Atomoxetine in children with ADHD and ASD. Studies could explore differences of Atomoxetine between high and low cognitive ability individuals with ASD, gender variability in treatment response, and age specific testing. This could help stratify the specific population of children with ASD that would benefit from Atomoxetine. As well, studies looking at including a comparison of Atomoxetine in conjunction with other therapies/medications or evaluating differences between variable versus fixed dosing of Atomoxetine in children with mixed phenotypes is warranted to assess differences in efficacy and safety. More testing inclusive to a wide range of ASD phenotypes and severity will be required to render these results generalizable to a broader ASD population. An area of consideration as well for future reviews of this subject is to include broader vocabulary for search terms such as “non‐stimulant” or “SNRI.” This broader search strategy may be able to find studies that include Atomoxetine under a group name such as “non‐stimulant” or “SNRI.” A narrower search strategy was implored in this review to focus on primary Atomoxetine studies and avoid a broad article search.

## CONCLUSIONS

Atomoxetine shows potential in treating common clinical symptoms of ADHD in children and adolescents with ASD given its inhibition action on the norepinephrine reuptake transporters. However, there is limited data available currently on Atomoxetine use in children and adolescents with ASD, suggesting the further need for research to establish its efficacy. This review emphasizes the need for more RCTs with larger studies for Atomoxetine in children and adolescents with ASD. Limited data does show Atomoxetine can be effective in treating common clinical symptoms of ADHD in the setting of ASD. Atomoxetine displays minimal side effects for children and adolescents with ASD with a favorable tolerability profile.

## AUTHOR CONTRIBUTIONS

**Nihit Gupta**: Conceptualization; data curation; formal analysis; investigation; methodology; project administration; supervision; writing—review and editing. **Daniel Boyes**: Formal analysis; investigation; methodology; writing—original draft; writing—review and editing. **Hunter Hanlon‐Taylor**: Formal analysis; investigation; methodology; writing—original draft; writing—review and editing. **Mayank Gupta**: Conceptualization; writing—review and editing.

## CONFLICT OF INTEREST STATEMENT

The authors declare no conflicts of interest.

## ETHICAL CONSIDERATIONS

The authors confirm that the ethical policies of the journal, as noted on the journal's author guidelines page, have been adhered to. This study did not involve working with human subjects and therefore was exempt from patient consent and ethics approval protocols.

## TRIAL REGISTRATION

This systematic review was performed according to PRISMA 2020 guidelines. The protocol was registered at PROSPERO: CRD42024491720.

## Data Availability

Data sharing not applicable to this article as no datasets were generated or analyzed during the current study.
